# Supplementary material for: Protein biogenesis machinery is a driver of replicative aging in yeast
Source: eLife. 2015 Dec 1;4:e08527. doi: 10.7554/eLife.08527 (PMC4718733; doi:10.7554/eLife.08527)
Supplement: Supplementary file 1. — DOI: http://dx.doi.org/10.7554/eLife.08527.044 [file elife-08527-supp1.doc]

Supplementary Materials for

**Protein Biogenesis Machinery is a Driver of Replicative Aging in Yeast**

Georges E. Janssens^1^*, Anne C. Meinema^2^*, Javier González^3^, Justina C. Wolters^5^, Alexander Schmidt^6^, Victor Guryev^1^, Rainer Bischoff^5^, Ernst C. Wit^4^, Liesbeth M. Veenhoff^1¶^, Matthias Heinemann^2¶^

* These authors contributed equally to this work.

¶ Co-corresponding authors:
m.heinemann@rug.nl (phone +31 50 363 8146, fax +31 50 363 4165), l.m.veenhoff@rug.nl (phone +31 6 527 24 855, fax +31 50 361 7310)

**This PDF file includes:**

Supplementary Text

Supplemental note 1: The explanation of the mathematical unmixing for

purification of aged cells page 2

Supplemental note 2: The rational behind the bead correction page 6

Supplemental note 3: Selection of the young time point reference sample page 7

Supplemental note 4: Network Analysis page 8

Supplementary References page 12

Supplementary Text

Supplemental note 1: The explanation of the mathematical unmixing for purification of aged cells

Here, we propose a mathematical solution for the separation of mixed end samples, resting on the idea that a system of linear equations can be solved when the number of unknowns equals the number of independent equations. In the case of our study, up to three unknowns are present in these samples; i.e. mother cells, dead cells (due to aging), and daughter cells. For the validation of the *unmixing* method described in this text, three samples have been chosen to be actual yeast cell cultures with known molecular profiles, which can be used as reference *pure-cell samples*. Manually mixing these *pure-cell samples* in known ratios reflects the aging experiment’s *mixed-cell samples* of mothers, dead cells, and daughters. Comparing the *unmixed abundance* values from the *unmixed-cell sample* to the *pure-abundance* values from *pure-cell sample’s* allowed us to assess the accuracy and validity of our method.

In order to acquire yeast cells of distinct cell profiles for empirical validation, samples were prepared coming from different phases of culture growth. These were log phase reflecting exponential growth (L), deceleration phase reflecting the shift in metabolism accompanying a nutrient depleted medium (D), and stationary phase, reflecting starved cells (S) (Figure 2–figure supplement 1A). The reader is referenced to specific terminologies used in this text in the list below. For illustrative purposes and for coherence with the experiments described in this study, the following stepwise description below will assume that we work with a single *molecule* (i.e. protein or mRNA) of interest, which is present in three *mixed-cell samples* that contain three cell types.

Terminology:

***Unmixing:*** The mathematical separation of *mixed-cell samples* into *unmixed-cell samples*, based on the known fractional contribution of cell types within the *mixed-cell sample* (stored in an *unmixing matrix*).

***Pure-cell sample***: A sample containing only one type of cell. This is only empirically available for the *unmixing* validation experiment for the samples of log phase (L), deceleration phase (D), and stationary phase (S).

***Mixed-cell sample***: a sample contained a known mixture of various cell types. For our aging studies these cell types correspond to mother, dead, and daughter cells. For our validation experiments these cell types correspond to log phase (L), deceleration phase (D), and stationary phase (S) cells of culture growth. The mix-cell samples are labeled as mixes (m) 1, 2, and 3.

***Unmixed-cell sample***: the sample resulting from the mathematical unmixing procedure, a mathematically purified sample that approximates the pure-cell samples.

***Molecule:*** in the case of this study, a protein or transcript.

***Pure abundance:*** the abundance value in a *pure-cell sample*, for a single *molecule* of interest.

***Mixed abundance***: the abundance value in a *mixed-cell sample*, for a single *molecule* of interest.

***Unmixed abundance:*** the abundance value in an *unmixed-cell sample*, for a single *molecule* of interest.

***Unmixing matrix:*** A matrix (W) used as input for the *unmixing* procedure. Contains the fractional composition of cell types (i.e. weights) within a *mixed-cell sample*. In the case of our aging study, these are objectively found by staining and flow cytometry, and represent live mothers, dead cells (due to aging), and daughter cells. In the case of our validation experiment, these were subjectively predetermined, to reflect a plausible situation of *mixed-cell samples* in the study, and *mixed-cell samples* were created accordingly.

We denote by **x** = (x_L_, x_D_, x_S_)^T^ the vector of *mixed abundances* in the three different *mixed-cell samples* composed of 3 types of cells (L, D, S), for a particular *molecule* of interest. These unknown values are to be solved by the mathematical *unmixing* procedure. We denote by **W** a 3 × 3 *unmixing matrix* containing in each row the fractional composition of the *mixed-cell samples* belonging to x_L_, x_D_ and x_S_. As fractional components of a *mixed-cell sample*, note that the sum of the entries of each row of **W** is one. We also denote by **m** = (m_1_, m_2_, m_3_)^T^ the vector of *mixed abundances* in three different *mixed-cell samples* (1, 2, 3), measured for a particular *molecule* of interest. Overall, we reason that **Wx = m**, (with **x** and **m** transformed for matrix multiplication) which can be formulated as


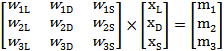
 (1)

where *w_ij_* represent the cell types *j* (i.e. L, D, S, or mothers, dead, and daughter cells for the validation experiments or aging experiments, respectively) present in the *mixed-cell sample* *i*. Under this formulation, the *mixed abundance* value of a *molecule* of interest is assumed to be a weighted sum of the *pure abundance* values of that *molecule*. These weights are the fractional composition of the *mixed-cell sample* (i.e. what percentage each cell type represents of the *mixed-cell sample,* Figure 2A). To illustrate this, consider that the *mixed-cell sample* m_1_ is an equal mixture of three *pure-cell samples* (i.e. the L, D, S cells are in a 1:1:1 ratio, therefore w_1L_, w_1D_, and w_1S_ would all equal 0.33), and that the *pure abundance* (x) is different in each cell type (L, D, S). Then we would have m_1_ = 0.33x_L_ + 0.33x_D_ + 0.33x_S_. Solving the equation (i.e. finding the abundance value of each x_L_, x_D_, and x_S_), would require two other linearly independent equations, each with a unique row entry in the *unmixing matrix* (**W**) and unique *mixed abundance* values (**m**).

In the context of our aging study, the *pure abundance* of *molecules* in the mother, dead, and daughter cells are unknown, and the goal of our *unmixing* method is to propose mathematically estimated *unmixed* *abundances* based on **W** and **m**. However, we remark that we refer to the *unmixed abundances* resulting from the mathematical *unmixing* procedure as being ‘estimated’ rather than ‘pure’, because in empirical applications there is inevitably noise-perturbed data coming from imperfect conditions. To address this we assume that we observe **m_ε_ = m + e** where **e** accounts for the noise associated to empirical studies. Likewise, the *unmixing matrix* **W** may contain weighted entries that contain highly similar fractional compositions of *mixed-cell samples*. In a theoretical case, this would pose no problems, however, in the context of experimental noise **e**, the system becomes increasingly difficult to correctly solve. Therefore, when **e** ≠ 0 the solution ***x̄*** (an estimated version of **x**) to the available system **m_ε_ = W*x̄***, differs from the target vector **x**. When considering the particular fractional row entries of **W** as possibly affecting the ability of the system to be solved, the difference between ***x̄*** and **x** can be theoretically bounded by


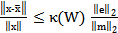
 (2)

where for ||e||_2_ the typical Euclidean l^2^ norm applies (i.e. being a positive value in Euclidean space) and where κ(**W**) is the condition number of W (i.e. a measure of the stability of the solution of the system) [1]. In our empirical case, we assume that **e** is kept at a minimum, as is observed by the high correlation between replicate measurements of our proteome and transcriptome data. However, matrices with a large condition number κ(**W**) (i.e. an *unmixing matrix* without clearly distinct weight entries) might nonetheless produce ***x̄*** solutions far from **x**. We note that in equation (2) if **e** is exactly equal to 0, even large values of κ(**W**) would pose no problems to correctly solve the system. Nonetheless, in empirical situations the constant κ(**W**) acts as an amplification of the error in the *unmixing* procedure. Therefore, a strategy to reduce the impact of a large condition number of **W** is needed, which satisfies that κ(**W**) is greater than 1.

To achieve this, we replace the system **m_ε_** = **W*x̄*** by a surrogate whose solution is close to the original one but with a much smaller condition number. In particular, we obtain the estimate ***x̄­­­­^*^*** of ***x̄*** by solving


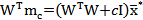
 (3)

where **W***^T^* is the transposed matrix of **W,** **I** is the identity matrix, *c* is a constant greater than 0 (i.e. the factor which contributes to the surrogate), and ***x̄^*^*** is the solution. In our unmixing processing *c* has been fixed to 0.01. We note that the properties inherent to solving linear systems using this approach are well appreciated and readily implemented in literature [1].

Equation (3) solves potential instability problems in the solution of the system. However, it does not take into account that in real applications the entries of the vector **m** should result in positive values since they correspond to the abundance of a molecule (i.e. a protein or transcript). Therefore, the solution to the system is found by using the Lawson-Hanson algorithm for non-negative least squares [2] [3], which attains a solution in the positive space (i.e. effectively setting any possible negative values to zero). In the case of our aging dataset, these generally occur for less than 10% of the data and are subsequently converted to “unsolvable” (i.e. NA), to have the time series of the molecule in question undergo further quality checks (see methods, mathematical *unmixing*). Following *unmixing*, a standard normalization of the datasets is required after solving the system in order to compare data independently *unmixed* (i.e. different time points).

To empirically validate the above *unmixing* method we used the cell cultures described above coming from different phases of growth (L, D, and S) to generate *pure-cell sample* profiles which would serve as references for the ‘correct’ solution to the system. Following this, we manually mixed these *pure-cell samples* into different *mixed-cell samples*, in the L:D:S ratios of 1:1:1, 3:7:10, and 6:3:1, to generate the three equations required for the *unmixing*. We generated ‘omics’ profiles for the three *pure-cell sample*s and three *mixed-cell samples* using targeted (SRM) proteomics measuring a selection of 207 metabolically related proteins, shotgun proteomics measuring abundances of 1942 abundant proteins, and RNA-seq measuring abundances of 6686 transcripts. With these profiles, we proceeded to *unmix* the *mixed-cell samples* into *unmixed-cell samples,* in order to compare the *unmixed abundances* to the *pure abundances* present in the *pure-cell samples*.

Figure 2–figure supplement 1C shows values of a representative set of 10 proteins coming from the 207 measured in the targeted SRM proteomics data, illustrating the ability of the *unmixing* method to approximate the *pure abundance* values from the *mixed abundance* values. Although as anticipated an error is introduced into the data (i.e. see Figure 2–figure supplement 1B), we found that our selected mixes (which represent a difficult case in mixing ratios, i.e. having an *unmixing matrix* with a large κ(**W**)), were able to retrieve the original proteomic profiles of each protein with an error of on average only 15.2% (measured using the Euclidean norm of the vectors of abundance values). In total, 179 proteins of the 207, representing 86.47% of the data, had an error less than 20%. A scatter plot of all 207 proteins, as *unmixed abundance* vs *pure abundance* illustrates the method’s ability to closely match the *pure abundance* values (Figure 2–figure supplement 1C).

When comparing the *unmixed abundance* values vs the *pure abundance* values generated by the shotgun proteomics, we found the Pearson correlation to be as high as 0.989, 0.992, and 0.993, for Log, Deceleration, and Stationary phase samples, respectively, in the log_2_ scale. In this case almost all proteins were recovered with less than 20% relative error (Figure 2–figure supplement 2A). The mRNA-seq transcriptome proved to have slightly less precise results than the shotgun proteome, but nonetheless provided favorable *unmixed abundance* values, with Pearson correlations of 0.945, 0.956, and 0.801 for Log, Deceleration, and Stationary phase samples, respectively, in the log_2_ scale (Figure 2–figure supplement 2B).

Based on the above we conclude that this method can be used in our study to mathematically *unmix* our *mixed-cell samples*, i.e. specifically to phenotype aging yeast mothers with minimal physical intervention resulting in an unprecedented mathematical purity of the population. Furthermore, this method has the potential for applications far beyond the scope of yeast replicative aging, to any rare sample difficult to enrich or purify. The method provided here is simple to use, has strong mathematical foundations, and has been demonstrated to be robustly applicable to a range of measurement techniques (i.e. targeted proteomics, shotgun proteomics, and RNAseq transcriptomics).

Supplemental note 2: The rational behind the bead correction

During pilot studies for this research and while studying protein extractions and resulting proteomes, we noticed that the raw datasets of samples containing beads had a consistently altered proteomic profile, different from identical samples that did not contain beads. We therefore tested for the possibility that somewhere in the pipe-line of sample processing the proteomic profile was altered due to biological responses occurring in the cells, or perhaps that this difference was chemically/physically related (i.e. due to interactions of extracted proteins with the bead surface). Testing samples were generated at different steps of biotinylation, bead labeling, harvesting, and stress inducing conditions, and samples were also produced which were simply cells mixed with beads. We found that the presence of beads alone within a sample was enough to offset the proteins detected in the sample, while that the process of bead labeling itself or the environmental stress applied yielded proteomes that bared little resemblance to our bead-containing samples (Figure 2–figure supplement 4A). Varying the numbers of beads present within the samples did not impact the proteome measured over a range of ratios relevant to our experiments i.e. our aged samples, which have a slight increase of bead/cell-ratio over time (Figure 2–figure supplement 4B, C, D, and E).

To address this effect of the presence of the beads, we calculated a correction factor that could be applied to normalized data, based on the difference between protein profiles of cells labeled with beads and cells not labeled with beads. For this, we used the ‘mother enriched fraction’ (Mix 2) (Figure 2–figure supplement 3A) sample of our column from the 0 hour (i.e. t0) time point, which contained cells in the presence of beads, compared to the ‘effluent fraction’ (Mix 3) from the same time point (t0) (Figure 2–figure supplement 3A). This ‘effluent fraction’ is from the same column as the ‘mother enriched fraction’, and therefore contained identical cells having essentially undergone the same processing but without having beads.

By subtracting the abundance value of a protein in the bead-containing sample (t0, Mix 2) from the abundance value of a protein in the sample not containing beads (t0, Mix 3) we obtained a correction factor to accommodate this bead-specific protein loss. The resulting vector of all correction factors for all proteins could be applied to each shotgun proteome library that contained beads as a way to redress the specific protein loss due to the presence of beads in the sample. We term the vector of correction factors for each protein the ‘correction factor-vector’.

Consistent with the idea that a predictable alteration of the proteome was occurring, the correction factor vectors between replicates had a Pearson correlation greater than 0.95. We therefore subsequently averaged the correction factor vectors between replicates to apply a single factor per protein to the raw data of each replicate’s sample that contained beads in the shotgun proteome (22 out of the 61 proteomes; see Figure 1-source data 1 Tables S2.2 a and b for dataset with correction, and Figure 1-source data 1 table S2.f for the correction factor-vectors). As the bead loss effect was not biologically based, the correction factor vector was applied prior to the mathematical unmixing procedure. Should negative abundances result from the correction, these were addressed in the unmixing algorithm, which effectively set these values to “unsolvable” during processing.

Supplemental note 3: Selection of the young time point reference sample

The aim of our study was to find casual factors in aging on a genome wide scale, and it was therefore important for us to maximize true positive aging related changes in our data and to minimize false positives, at the potential cost of increasing false negatives. Our aims were likewise to produce final datasets of raw abundance values that could be used to generate comparisons between proteomes and transcriptomes in order to see how correlations of the datasets change in time. To accommodate these requirements, we devised a strategy to select an appropriate reference sample as follows.

It is clear that an unprocessed exponentially growing yeast sample cannot be the reference sample, since it did not undergo the similar processing steps as the aging time course samples. Likewise, time points early after the loading procedure may still be in a responsive mode reflecting the biotinylation procedure and column loading which would have just taken place. Therefore, we aimed to choose a time point in our time series that we thought reflected yeast in a young stage of life, which had recovered from any potential handling related changes resulting from biotinylation and column loading. This corresponded to our 7.8 hour time point, the first time point in common between our replicate datasets, and a time point at which the cells had undergone no more than 5 divisions.

A standard method for subsequent analysis of the data would be to consider later time points relative to this one, and to convert the data to fold changes. The benefit of this is that any biological changes resulting from processing steps occurring after the loading procedure, i.e harvesting and purification of the samples, would be accounted for since all samples will have undergone similar treatment. This process, however, renders the data in a fold change format, and does not fulfill our requirement to have raw abundance datasets.

Therefore, we devised a strategy to project the aging related fold changes relative to our 7.8 hour time point, onto the raw abundance profiles of unprocessed, logarithmically growing cells. This would result in a time series of raw abundance values of aging related changes. To accomplish this, we used the same method previously applied for bead-effect correction (see supplemental note 2), but considered the difference present when comparing our unprocessed sample compared to our 7.8 hour sample, as the correction factor required to remove any background present from the harvesting procedure. Similarly to as with the bead correction, these factors correlated highly between replicates, and were therefore averaged prior to being applied to the time series. Having mathematically unmixed datasets at this stage of data processing, this method was applied individually for both mothers and daughters (see Figure 6-source data 1 Table S2.4 a and b, and Figure 6-source data 2 S3.4 a and b, for the proteome and transcriptome, respectively). Following this, as was previously implemented in the mathematical unmixing methods, should the resulting standardized data contain negatives, a data quality criteria was applied: at least three time points from those measured in the experiment (7.8 hour to 72 hour) were required to contain positive entries, otherwise the gene was removed from the dataset. Finally, as was also implemented in the mathematical unmixing methods, in cases that passed the quality criteria but still contained one or more negatives in the time series, the data was linearly interpolated by neighboring time points [4]. Following this, all datasets were normalized to 1 million for both proteomes and transcriptomes, and analysis of the data was performed with the 7.8 hour time point as the young time point of reference, for either correlations on the raw dataset, or fold change enrichment analyses.

Supplemental note 4: Network Analysis

To infer the high-level directional networks (Figure 6B, Figure 7–figure supplement 4B) and find causal relations, six data analysis steps (Figure 6–figure supplement 1A) were undertaken, as expanded upon below. Briefly, these were: 1. Starting from the replicate datasets, the gene expression time series of both the transcriptome and proteome were filtered to remove flat and/or noisy profiles using the R package GPREGE [5]. 2. The gene product networks (i.e transcriptome or proteome) were generated, based on the gene profiles of the respective time course data sets, using the R package GeneNet [6,7]. This included generating an undirected network by calculating the partial correlation among gene profiles (Figure 6–figure supplement 1B and C, Figure 7–figure supplement 4A). 3. Following this, a directed network was generated from the undirected network, based on an assessment of a gene profile’s ability to predict another gene profile (Figure 6–figure supplement 1D and E, Figure 7–figure supplement 4A) [6,7]. 4. The nodes in the network were clustered together, using the method in [8] using the R package igraph (Figure 6A, Figure 7–figure supplement 4A) [9]. The causal in/out connection among genes were calculated for all the network clusters and listed in a direction matrix (listed in Figure 6-source data 1 table S7). 5. A high-level directional network was generated, where the clusters are plotted in order of their causal ranking by drawing the direction matrix as arrows between the clusters. 6. A sensitivity analysis was made to determine the optimal sparsity of the networks and the cut-off for the partial correlation among gene profiles.

1) Data filtering

As input for the inference of the network, we used the non-fitted transcriptome and proteome data from replicates R1 and R2 (Figure 1-source data 1 Table S2.4 a and b, Table Figure 1-source data 2 S3.4 a and b). The young 7.8h time point was chosen as a reference sample (see supplemental note 3). All 12 time-points from 7.8 to 72.3h were used. In cases where a time point was missing in one of the two replicates, it was linearly interpolated using neighboring data points with the ‘approx’ function from the R-package zoo [4].

All gene expression profiles (time-series of either proteins or transcripts) were ranked using the R-package GPREGE [5]. In short, this software estimates the continuous trajectory of gene expression by means of Gaussian process regression and uses the ratio of the marginal likelihood between a model in which a curve is fitted to the data (Hi, differentially expressed) and a flat baseline model (H0, non-differentially expressed). By defining a threshold for likelihood ratio Hi/H0, we could filter the data by controlling the removal of flat and/or noisy expression time-series data. To select the most appropriate threshold for our data, we performed a sensitivity analysis (together with other parameters of the network reconstruction method, see below) in which the threshold for Hi/H0-ratio was studied from 0.1 to 0.3 for the proteome data and from 1 to 2 for the transcriptome (see below).

2) Generation of undirected networks

Undirected networks were generated for the proteome and transcriptome gene expression time series (i.e. gene profiles) by using the GeneNet R-Package [7]. An edge between nodes in the undirected network indicates a pairwise connection between two gene profiles, accounting for the influence of the rest of the network. Only nodes that have related gene profiles (based on partial correlations), as distinguished from indirectly related gene profiles (based on simple correlations), are connected in the network (Figure 6–figure supplement 1B). In short, the undirected network was generated by calculating the partial correlation among gene profiles, found by means of a regularized estimator of the precision matrix (inverse of the correlation) (v) (Figure 6–figure supplement 1B) [10]. This takes into account the dynamic and interdependent nature of the data. This step is important to find the causal relations in the next step

The cut-off for the partial correlation, that provides a measure of the sparsity of the final network, was varied from 0.4 (very dense network, many connections) to 0.95 (very sparse network, a few connections) in the sensitivity analysis (see step 6 below).

3) Generation of the directed networks

Once the undirected network was calculated, the directionality between two nodes in the network was obtained only for related gene profiles.. The directionality of the arrows between two nodes was found by accounting for the relative reduction in the variability between them. This is achieved by multiple testing of the standardized partial variances between nodes, that is, the variances once the effect of the associated neighboring nodes have been removed (Figure 6–figure supplement 1D). For each pair of connected gene profiles, the standardized partial variance was compared resulting in the direction of the connecting arrow going from the gene with the larger standardized partial variance to the gene with the smaller standardized partial variance. Basically, for a node with a lower standardized partial variance, much of its variability is explained by the nodes associated to it, while for a node with a high standardized partial variance, less of its variability is explained by the nodes associated to it. This makes a node with a high standardized variance causal over a node with a low standardized variance. This provides an ordering of the nodes, which imposes directionality on the network edges. A directed edge from node ‘m’ to node ‘n’ is called an outgoing connection form, and an incoming connection for n. The connections are interpreted as ‘m causes n’, since the undirected network link between m and n shows an actual relation, and the directionality between the nodes shows that much of the variability of n is explained by m (Figure 6–figure supplement 1D). This shows the influence of one node on the other

4) Clustering of the network nodes

The network clusters were found by searching for optimized network communities that are highly intra-connected and less inter-connected. Specifically, the clusters of the networks were obtained by means of the method in [8] using the R package igraph [9]. This method finds clusters based on the topology of the network by maximizing the global modularity (requiring nodes in a cluster to have a larger degree of connectivity with other nodes in the same cluster than with the rest, Figure 6A, Figure 7–figure supplement 4A). The algorithm is based on a geometrical interpretation of the adjacency matrix A (a_ij_=1 if nodes i and j are connected, a_ij_=0 otherwise) that is typically used to represent the networks. The sum of the incoming and outgoing directional connections among nodes per cluster are listed in a direction matrix (Figure 6-source data 1 Table S7)

5) High-level directional networks

Rather than doing a single-node causal-effect interpretation of the networks, our goal was to provide a general understanding of the high-level phonotypical changes occurring in time in the dataset. To obtain such interpretation we defined criteria to sort out the clusters in a virtual, but coherent with the experimental, time 'causality' scale.

A network cluster is labeled as high-level causal if it has the highest ratio of outgoing over incoming links, whereas it is labeled as responsive otherwise. A high-level causal cluster contains many gene profiles that can predict gene profiles in other clusters. We interpret these causal gene profiles to belong to early age changes, while responsive clusters belong to later in life changes. The number of node connections between clusters and the direction of the links between the clusters define the magnitude and direction of the connection between clusters. To complete the interpretability of the network clusters, GO-term enrichment analysis was performed. The *p*-values associated to the significant GO terms were obtained using the R packages GOstats [11] and org.Sc.sgd.db [12].

6) Sensitivity analysis: choosing the most relevant biological network

The threshold to filter non-expressed/noisy genes (Hi/ H0) and the sparsity of the network (α) are needed to uniquely identify the proteome and transciptome networks. Although a statistical model selection criterion could have been used here to choose the optimal parameters, we decided to follow a biological-driven approach able to enhance the final high-level interpretability of the results.

Accordingly, we selected the networks according to two different criteria:

1. A high degree of causality (DoC), which is defined as DoC = Me({r_ij}), for ij=1,...,n where n is the number of clusters in the network, Me represents the median and r_ij represents the ratio of the outgoing over the incoming connections between clusters i^th^ and j^th^. The assumption here is that a network with higher DoC shows a higher degree of causality, being that the edge distributions between clusters are more different to what one would expect in a pure random case.
2. A low median *p*-value of assigned GO-terms (Mpv-GO), which enhances network’s cluster interpretability.

For the proteome and the transcriptome datasets, 24 and 30 networks were generated, respectively, varying the cut-off for the likelihood of gene expression (Hi/H0) and the cut-off for partial correlation among genes (α). From this, we excluded three networks (1 in the proteome and 2 in the transcriptome networks, see Figure 6-source data 1 Table S7.1 and S7.2, grey text,) that showed a high degree of partitioning, i.e. many clusters with less than 10 nodes (i.e. more than 50%), having a low cluster enrichment for GO-terms (*p*-value >10^-3^). We selected, from the 6 networks with the highest DoC, the 3 with the smallest Mpv-GO (See Figure 6-source data 1 Table S7.1 and S7.2). To check the coherence of both criteria we submitted the cluster gene lists to the Gene Functional Classification Tool of DAVID version 6.7 (See, GO term selection and annotation) [13]. The three candidate networks for transcriptome and the proteome were compared by using the cluster GO summaries clustering ordering. These networks (in both the proteome and transcriptome cases) resulted in highly similar ordering of causal events. The network with the lowest *p*-value was selected for visualization in the publication (see Figure 6-source data 1 Table S7.1 and S7.2, bold text). The sum of incoming and outgoing node cluster links for the selected networks is available in the direction matrix (Figure 6-source data 1 Table S7.3 and S7.4).

Supplemental references

[1] L.N. Trefethen, D. Bau III, Numerical linear algebra, 1997. doi:10.1137/1.9780898719574.

[2] C.L. Lawson, R.J. Hanson, Solving least squares problems, 1995. doi:10.2307/2005340.

[3] K.M. Mullen, I.H.M. van Stokkum, nnls: The Lawson-Hanson algorithm for non-negative least squares (NNLS), (2012). http://cran.r-project.org/package=nnls.

[4] A. Zeileis, G. Grothendieck, ZOO: S3 Infrastructure for Regular and Irregular Time Series, J. Stat. Softw. 14 (2005) 1–27.

[5] A.A. Kalaitzis, N.D. Lawrence, A simple approach to ranking differentially expressed gene expression time courses through Gaussian process regression., BMC Bioinformatics. 12 (2011) 180. doi:10.1186/1471-2105-12-180.

[6] R. Opgen-Rhein, K. Strimmer, From correlation to causation networks: a simple approximate learning algorithm and its application to high-dimensional plant gene expression data., BMC Syst. Biol. 1 (2007) 37. doi:10.1186/1752-0509-1-37.

[7] J. Schaefer, R. Opgen-Rhein, K. Strimmer, GeneNet: Modeling and Inferring Gene Networks, (2015). http://cran.r-project.org/package=GeneNet.

[8] M.E.J. Newman, Finding community structure in networks using the eigenvectors of matrices, Phys. Rev. E - Stat. Nonlinear, Soft Matter Phys. 74 (2006). doi:10.1103/PhysRevE.74.036104.

[9] G. Csardi, T. Nepusz, The igraph software package for complex network research, InterJournal. Complex Sy (2006) 1695. doi:citeulike-article-id:3443126.

[10] J. Schäfer, K. Strimmer, A shrinkage approach to large-scale covariance matrix estimation and implications for functional genomics., Stat. Appl. Genet. Mol. Biol. 4 (2005) Article32. doi:10.2202/1544-6115.1175.

[11] S. Falcon, R. Gentleman, Using GOstats to test gene lists for GO term association, Bioinformatics. 23 (2007) 257–258. doi:10.1093/bioinformatics/btl567.

[12] M. Carlson, org.Sc.sgd.db: Genome wide annotation for Yeast, (n.d.).

[13] D.W. Huang, B.T. Sherman, R.A. Lempicki, Systematic and integrative analysis of large gene lists using DAVID bioinformatics resources., Nat. Protoc. 4 (2009) 44–57. doi:10.1038/nprot.2008.211.
